# Editorial

**Published:** 1869-12

**Authors:** 


### Editorial.

---

CRIMINAL ABORTIONS.—The deaths from criminal attempts to procure abortions are disgracefully frequent. Many of them are never known to the public; and, yet, the number that are detected and published are sufficient to make any civilized community blush with shame. Within the last ten days, two cases have occurred in this city, under such circumstances as to attract the attention of the legal authorities, and to cause investigation. In the first case, the girl was an inmate of a house of ill-fame, and the operation is alleged to have been performed by Henry R. Stratford, claiming the title of doctor. The other appears to have been a servant girl, seduced by some wretch and abandoned. The alleged operator, in her case, was Duncan MacRae, a reckless pretender, who for many years has advertised his ability to cure cancers and all other tumors, etc. In both cases, death followed the operation, in a few days. From the evidence before the Coroner's Jury, it would appear that producing abortions constituted a prominent part of the practice of both the misnamed doctors. They were promptly arrested by the police, have been indicted by the Grand Jury, on a charge of *murder*, and are now awaiting their trial in the Circuit Court. We think their indictment for *murder* is a mistake. Several such indictments have been found against different parties in this city before, but never has one been convicted, no matter how positive the evidence or how mercenary the crime. Neither is there likely to be any convictions on such indictments. The plain fact that the criminals acted without any malice or ill-will towards their victims, but, on the contrary, with the consent and at the solicitation of the latter, with the ability of counsel to present a plausable pretext that the victim died, not from the abortion, but from some accidental inflammation, will always render it difficult to procure a conviction of murder. It seems to us better to try all such cases on the simple charge of producing the abortion, on which they would be certainly convicted, and then inflict the full penalty allowed by the law. A few prompt convictions, even if the penalty was inadequate in comparison with the magnitude of the crime, would do more to suppress the horrid practices of the abortionist than any number of indictments for murder.

---

Clinical Instruction to Mixed Classes.—It seems that our professional brethren in the colleges and hospitals of Philadelphia, that city of *brotherly love*, have had their sober sense of propriety and their inate modesty rudely shocked by the appearance of a company of *female* medical students, at the Pennsylvania Hospital. So rude, indeed, has been the shock, that it has called forth a formal protest against such attendance of females in company with the males, signed by the faculties of the Pennsylvania University, the Jefferson Medical College, and the boards of physicians and surgeons of the several hospitals in that city.

The basis of the protest is as follows:—

1. Clinical instruction in practical medicine demands an examination of all the organs and parts of the body, as far as practicable; hence, personal exposure becomes, for this purpose, often a matter of absolute necessity. It cannot be assumed, by any right-minded person, that male patients should be subjected to inspection before a class of females, although this inspection may, without impropriety, be submitted to before those of their own sex. A thorough investigation, as well as demonstration, in these cases, so necessary to render instruction complete and effective, is, by a mixed audience, precluded; while the clinical lecturer is restrained and embarrassed in his inquiries, and must, therefore, fall short in the conclusions which he may draw, and in the instruction which he communicates.

2. In many operations upon male patients, exposure of the body is inevitable, and demonstrations must be made which are unfitted for the observation of students of the opposite sex. These expositions, when made under the eye of such a conjoined assemblage, are shocking to the sense of decency, and entail the risk of unmanning the surgeon, of distracting his mind, and indangering the life of his patient. Besides this, a large class of surgical diseases of the male is of so delicate a nature as altogether to forbid inspection by female students. Yet a complete understanding of this particular class of diseases is of preëminent importance to the community. Moreover, such affections can be thoroughly studied only in the clinics of the large cities, and the opportunity for studying them, so far from being curtailed, should be extended to the utmost possible degree. To those who are familiar with such cases as are here alluded to, it is inconceivable that females should ever be called to their treatment.

3. By the joint participation, on the part of male and female students, in the instruction and in the demonstrations which properly belong to the clinical lecture-room, the barrier of respect is broken down, and that high estimation of womanly qualities, which should always be sustained and cherished, and which has its origin in domestic and social associations, is lost, by an inevitable and positive demoralization of the individuals concerned, thereby entailing most serious detriment to the morals of society. In view of the above considerations, the undersigned do solemnly protest against the admixture of the sexes, at clinical instruction in medicine and surgery, and do respectfully lay these their views before the boards of managers of the hospitals in Philadelphia.

*November 15, 1869.*

It will be seen that the protestants make the whole matter a simple question of modesty or propriety. They utter no word against females studying or practicing medicine and surgery, but only against their studying and attending clinical instruction, in compony with the opposite sex. We are satisfied that the position thus assumed is untenahle. The *indelicacy*, the impairment of *respect* for the sex, and the danger of *disconcerting* the operator or clinical teacher, in his work, are all propositions that look formidable in prospective, but which vanish when brought practically to the test. The rule on which every every intelligent physician daily rests is, that whatever is *necessary* for the proper investigation of disease and the relief of suffering is neither indelicate nor improper. It is on this broad, plain rule that the profession has justified itself in dissecting dead bodies, in examining the sexual organs of the female, and in admitting students of either sex to all the necessary practises of the clinical wards of public hospitals. If a company of young men can witness examinations and operations on the sexual organs of the female, without indelicacy or impropriety, by what kind of logic can it be maintained that it is indelicate or improper for an audience of females to witness examinations and operations on the sexual organs of the male? And if such company may witness these things separately, why not together? Each company justifies itself separately by its motive, namely, that of gaining such knowledge of disease as will enable its individual members to alleviate human suffering and prolong human life. And if each is actuated by this motive, and this only, there will be neither more indelicacy or embarrassment in looking together, at one and the same suffering patient, than if they were an hundred miles apart. If actuated by this motive (and it is only this that can justify the presence of either sex at all), the presence of both sexes simply operates as a check to heedless and unnecessary exposures of the sick, and to the indulgence of a propensity to tell *smutty stories* in the lecture-room, which, we are sorry to know, some medical teachers possess.

The moment we admit that there is any question of indelicacy or impropriety in admitting students of either or both sexes to such examinations and operations as are necessary to fit them to practise the profession of medicine, surgery, and obstetrics intelligently and beneficially to the community, that moment we lay the foundation for the claim that each sex must have a doctor of its own gender, and the presence of a male doctor in the bed-chamber of a female patient becomes a sexual impropriety. We do not believe that even Philadelphia modesty and refinement is prepared for such a conclusion. The truth is, that for several years past, females have attended freely clinical instruction, in the same classes with the men, in some of the best hospitals in Europe and America. We understand they are now attending the Bellevue and other hospitals in New York; and when, on a recent occasion, some one offered a resolution opposing their attendance, it was promptly voted down by the male members of the class in Bellevue Hospital.

A few female students have attended regularly the clinics in the Cook County Hospital, in this city, in the same classes with the young men, for two or three years past. Although the clinical instructors of that institution are proverbially modest men, and some of them, at least, unmarried, we have heard no intimation that they had been disconcerted or in the least interfered with in the proper performance of their duties.

While, in the foregoing remarks, we repudiate the idea of indelicacy in the study of medicine, by either or both sexes, we by no means advise females to study medicine or practise it as profession. Of all the secular employments in civilized society, there are very few so little suited to the nature and necessities of woman. The labor, mental and physical, the exposures, at all hours, and in all places, the necessity of being ready for action night or day, in the mansion or the hovel, are all circumstances incompatible with the physical necessities und designs of the sex. We have no sympathy with the modern hub-bub about womans' rights and wrongs. But a few singularly constituted women will study medicine, and the quicker the profession everywhere cease to pay any special attention to them, either by favor or opposition, the sooner will they cease to attract attention or call forth newspaper notoriety.

---

EXTRACT of ergot for subcutaneous injection is thus made, from a formula recently published: Ext. ergot two parts, alcohol and glycerine, of each, seven parts.

---

HIPPOCRATES died at the age of 109; Galen lived to be 104; Solon and Thales 100; Zeno 98; Diogenes 88; Plato 94; Lycurgus 85; Sophocles more than 100; Asclepiades also Juvenal 100; Newton 85; Fontenelle 99; Buffon 81; and Voltaire 84.—*Med. Gazette.*

---

PRURITUS VULVA.—It is stated that pruritus vulva is often entirely cured by a lotion consisting of five parts of corrosive sublimate dissolved in 50 parts of alcohol. A teaspoonful of this solution is diluted with a pint of tepid water, and applied as a wash to the parts three times daily.—*St. Louis Medical Archives.*

---

WARTS.—The modern scientific name of this disorder is *Myrmekiasmos*, but the principal point is the surprising rapidity with which an extensive cluster of warts will disappear under the use of arsenic, without any local application whatever. Potassa fusa has been used locally. The warts should be saturated with a solution of equal parts of potassa fusa and water, until the horny growth is dissolved and the papillæ destroyed by this caustic.—*Medical Gazette.—St. Louis Med. Archives.*

### Mortality for the Month of October, 1869:—

CAUSES OF DEATH,

| | |
|---|---|
| Accident, drowned | 2 |
| " crushed by timber | 1 |
| " burned by kerosene | 2 |
| " excessive gymnastics | 1 |
| " by fall | 5 |
| " morphine | 1 |
| " run over by truck | 1 |
| " by railroad, | 3 |
| " thrown from wagon | 1 |
| " suffocation | 1 |
| " scalded | 1 |
| " injured by water pipe | 1 |
| Anus imperforate | 1 |
| Anguina | 1 |
| Apoplexy | 8 |
| " and pneumonia, | 1 |
| Ascites | 1 |
| Asphyxia | 2 |
| Births, premature | 28 |
| " still | 44 |
| " tedious | 1 |
| Brain, inflammation of | 4 |
| " softening of and cancer | 1 |
| Bronchitis & teething, | 1 |
| Cancer, scirrhus | 1 |
| " of mammary gland | 1 |
| Caecum, inflammation of and peritoneim | 1 |
| Cerebritis & paralysis, | 1 |
| Cholera infantum | 31 |
| Cirrhossis, hepatic | 1 |
| Convulsions | 45 |
| " puerperal | 2 |
| " and teething | 1 |
| Consumption | 44 |
| " and dropsy, | 1 |
| " and heart, valvular disease of | 1 |
| Croup | 21 |
| " diphtheretic | 2 |
| " membranous | 5 |
| Cranium, malformation of | 1 |
| Crusta lactia retrocession of, | 1 |
| Cyanch maligna | 2 |
| Cyanosis | 1 |
| Debility | 6 |
| " general | 2 |
| Delirium tremens | 3 |
| Diarrhœa | 20 |
| " and burns of body and arms, | 1 |
| " chronic | 9 |
| Diphtheria | 27 |
| " and scarlet fever, | 1 |
| Dropsy | 6 |
| Dysentery | 14 |
| " chronic | 3 |
| " acute | 1 |
| " typhoid | 1 |
| " and phrenitis, | 1 |
| Entero-colitis | 1 |
| " chronic | 1 |
| Enteritis | 8 |
| " acute | 1 |
| Epilepsy | 1 |
| Erysipelas | 1 |
| " and icterus | 1 |
| Fever, congestive | 2 |
| " puerperal | 3 |
| " remittent | 5 |
| " scarlet | 42 |
| " " malignant, | 1 |
| " " and diphtheria | 4 |
| " typhoid | 36 |
| " " and lungs, congestion of | 1 |
| " " and bowels, mortification | 1 |
| Gastritis | 3 |
| Gastro enteritis | 1 |
| Hemorrhage from fever sore | 1 |
| Hæmorrhage, internal from aneurism | 1 |
| Heart, disease of | 2 |
| " " and pneumonia | 1 |
| " " and intemperance | 1 |
| " hypertrophy of, | 1 |
| " organic disease of | 1 |
| " valvular disease | 3 |
| Hepatitis | 1 |
| Hydrocephalus | 6 |
| " acute | 6 |
| Inanition | 14 |
| Intemperance | 4 |
| Knee, white swelling of | 1 |
| Kidneys, Bright's disease of | 3 |
| " " and hemorrhage after confinement | 1 |
| Laryngitis | 2 |
| Liver, disease of, and dropsy | 1 |
| " abscess of | 2 |
| " induration of and dropsy | 1 |
| " cirrhosis of | 1 |
| " paralysis of | 2 |
| Lungs, congestion of | 5 |
| " paralysis and brain congest'n | 1 |
| " hemorrhage of | 1 |
| Manslaughter | 1 |
| Measles | 2 |
| " and diarrhœa | 1 |
| Metro-peritonitis, puerperal | 2 |
| Meningitis | 2 |
| " cerebro-spinal, | 1 |
| " tubercular | 2 |
| Metritis, puerperal | 1 |
| Neck, cancer of | 1 |
| Nephritis | 1 |
| Necrosis | 1 |
| Old age | 12 |
| Occipital bone, depression of | 1 |
| Paraplegia from injury to back | 1 |
| Paralysis | 2 |
| " from intemperance | 1 |
| Pericarditis following measles | 1 |
| Peritonitis | 3 |
| Phlebitis | 1 |
| Phrenitis | 2 |
| Pneumonia | 16 |
| " typhoid | 3 |
| " and cholera infantum | 1 |
| Pyæmia | 3 |
| Scrofula | 1 |
| Spleen, tubercular enlargement of | 1 |
| Stomach, cancer of | 1 |
| Suicide | 3 |
| Tabes mesenterica, | 24 |

| | | | | | |
|---|---|---|---|---|---|
| Teething | 7 | Trismus | 1 | Whoop.-cough & brain congestion | 1 |
| " and cholera infantum | 2 | Uræmia | 1 | " " lungs congestion of | 1 |
| Tetanus | 1 | Uterus, cancer of | 2 | Unknown | 1 |
| Throat, ulcerated sore, | 1 | " hemorrhage of, "childbirth" | 1 | | |
| Tongue, gangrene, malignant | 1 | Whooping-cough | 10 | Total | 597 |
| | | " " bronchitis | 1 | | |

COMPARISON.

Deaths in Oct., 1869, 597 | Deaths in Oct., 1868, 449 | Increase, 148

Deaths in Sept., 1869, 814 | Decrease, 217

AGES.

| | | | | | |
|---|---|---|---|---|---|
| Under 1 | 165 | 20 to 30 | 64 | 70 to 80 | 19 |
| 1 to 3 | 131 | 30 to 40 | 41 | 80 to 90 | 4 |
| 3 to 5 | 44 | 40 to 50 | 19 | 90 to 100 | 1 |
| 5 to 10 | 43 | 50 to 60 | 24 | | |
| 10 to 20 | 29 | 60 to 70 | 13 | Total, | 597 |

| | | | | | |
|---|---|---|---|---|---|
| Males, | 322 | Females, | 275 | Total, | 597 |
| Single, | 471 | Married | 126 | Total, | 597 |
| White, | 594 | Colored, | 3 | Total, | 597 |

NATIVITY.

| | | | | | |
|---|---|---|---|---|---|
| Bohemia, | 1 | England, | 12 | Sweden, | 15 |
| Canada, | 9 | France, | 2 | Scotland, | 2 |
| Chicago, Native, | 100 | Germany, | 74 | Switzerland | 1 |
| Chicago, Foreign, | 216 | Holland | 2 | Unknown, | 2 |
| U. S., other parts, | 87 | Ireland, | 57 | | |
| Denmark, | 5 | Norway, | 12 | Total, | 597 |

MORTALITY BY WARDS FOR THE MONTH.

| Wards. | Mortality. | Pop. in 1868. | One death in |
|---|---|---|---|
| 1 | 7 | 9,094 | 1299 1-7 |
| 2 | 23 | 13,074 | 568½ |
| 3 | 21 | 15,076 | 718 |
| 4 | 21 | 17,796 | 847½ |
| 5 | 51 | 16,033 | 314⅓ |
| 6 | 26 | 13,083 | 503 1-5 |
| 7 | 50 | 25,492 | 510 |
| 8 | 59 | 15,813 | 268 |
| 9 | 32 | 19,297 | 603 |
| 10 | 12 | 12,925 | 1077 |
| 11 | 20 | 14,340 | 717 |
| 12 | 85 | 17,485 | 205¾ |
| 13 | 19 | 11,164 | 587½ |
| 14 | 34 | 14,839 | 436½ |
| 15 | 39 | 21,078 | 702½ |
| 16 | 18 | 15,465 | 396½ |

| | Mortality. |
|---|---|
| Accidents, | 20 |
| County Hospital, | 18 |
| Immigrants, | 14 |
| Mercy Hospital, | 10 |
| Massasoit House, | 1 |
| Manslaughter, | 1 |
| Protestant Orphan Asylum, | 2 |
| St. Joseph Orphan Asylum, | 9 |
| Steamer S. Van Epps, | 1 |
| Soldiers' Home, | 1 |
| Suicide, Poison, | 3 |
| Total, | 597 |

---

THE extract of Calabar bean when instilled into the eye produces contraction of the pupil, but when injected hypodermically, it produces dilatation of the pupil. It has been found to be the best antidote for atrophine. Epilepsy treated by it is not removed or modified. It has given good results in the treatment of chorea.—*Richmond and Louisville Med. Journal.*

### ARSENIC IN DISEASES OF THE SKIN.

Thomas Hunt, F.R.C.S., Surgeon to the London Western Dispensary for Diseases of the Skin (*Journal of Cutaneous Medicine*), speaks of arsenic as follows:—We have a remedy for at least one-half of the diseases to which man is subject. Arsenic, therefore, if rightly used, is adapted to the treatment of six out of every seven cases of chronic skin disease which the physician is asked to relieve. Supposing the preparation to be Fowler's solution, he states that it should be carefully administered. *First.* It should be given in divided doses—three doses in 24 hours, simply to avoid an unnecessarily large dose. *Second.* It should be diluted with pure water, or if the case require the influence of antimony, the vinum antimonii, say 35 minims, may be combined with five minims of the liquor arsenicalis, and this may be taken diluted three times a day. *Third.* This dose should be taken with, or immediately *after*, a meal, in order that, being mixed with the patient's food, it may find a ready entrance into the blood, and that the bare possibility of its irritating the mucous membrane of the stomach or bowels may be avoided. Not that there is any danger of mischief, but the patient, aware that he is taking arsenic, may be disabused of all fanciful or imaginary suffering of this kind. *Fourth.* Arsenic acts very slowly, and, therefore, it is best to begin with an average dose, say five minims of Fowler's solution; and this should be increased, not day by day, as was the practice 30 years ago, but two, three, or four weeks should be allowed to elapse before any necessity can exist for augmenting the dose. If an active or severe inflammation of the tunica conjunctiva in *both* eyes should be set up, then the medicine is not to be abandoned in a panic, but the dose may be reduced to four minims, and in a week or ten days the conjunctiva will be less inflamed. During the first week, if the patient has been properly prepared for the course, the disease of the skin will show some amendment; it will be shorn of its strength, and from this time the cure will be easy enough, although it may take weeks, or even months, to effect it entirely. The health of the patient must be closely watched, and the secretions carefully sustained. Above all, the bowels must not be allowed to act sluggishly. In many cases, a full dose of calomel and comp. colocynth pill will be required two or three times a week; and these doses are sometimes essential to the cure.

If the legs, feet, or abdomen become puffy or œdematous, the urine being scanty or turbid, the case will not progress well until the kidneys are aroused to vigorous action by full doses of spiritus ætheris nitrosi and acetate of potash largely diluted with water. Young children bear arsenic well, and girls about the age of puberty generally bear and require larger doses than adults of either sex. A child of one year old will require about half the dose proper for an adult. Finally, he recommends freshly prepared Fowler's solution, of a light color, as the red lavender, which for some foolish reason is generally mixed with the solution, often becomes stale and nauseous. The best way of measuring the dose is to mix a drachm of Fowler's solution with seven drachms of water, giving 40 minims as the proper dose, representing five minim doses of the original solution; by measuring 40 minims each time, the patient has a better chance of getting the right dose than in the uncertain bulk of *drops* and *spoons.—Medical Record.*

---

Favorable Result of Transfusion.—*The Boston Medical Journal*, quoting from the *Medical Times and Gazette*, gives the following encouraging account of the result of transfusion:

Professor Landois, of the University of Griefswald, who has interested himself much in the subject of transfusion, after giving a critical account of the most recent publications on the subject, thus sums up, in a recent number of the *Wien. Med. Woch.*, the results that have hitherto been obtained:—1. Transfusion has been performed 99 times in cases of hemorrhage, in eleven of which cases, no successful result was even possible. Of the remaining 88 cases, 65 were attended with success, 20 were unsuccessful, and in 3 the result was doubtful. 2. It has been performed 12 times in cases of acute poisoning, one of these being hopeless. In three the results were favorable, and in eight unfavorable. 3. For various forms of disease attended with exhaustion, it has been resorted to 43 times, the most unfavorable prognosis having been frequently delivered. In these, the results were favorable in 12, unfavorable in 21, and doubtful in 9, while in one case it was a mere desperate experiment. Professor Landois observes that these statistics speak very satisfactorily for transfusion, and that the results would be far more favorable if this almost harmless operation were not usually driven on to the last minute.—*St. Louis Medical Archives.*

---

The Minute Structure of the Pancreas.—It is generally believed that the structure of the pancreas is identical with that of the salivary glands. M. Giannuzzi has made this organ the subject of microscopic research, and points out the following differences:—"The excretory ducts of the pancreas have very thin walls, which are covered interiorly by a cylindrical epithelium. They have not the same connections with the secreting vesicles as in the salivary glands, but they establish around them a network composed of very fine tubes, which have no epithelium, and which surround the pancreatic cellules in their meshes. This network may be compared to that of the biliary ducts of the liver. The network of the excretory ducts of the different vesicules which form the same glandular lobule have connections between them, and constitute a common network. The terminal ramifications of the bloodvessels of the pancreas follow, in general, the tracts of the pancreatic ducts. The pancreatic vesicules are without The pavement epithelium of the vesicules is formed of flattened cellules with a nucleus and prolongation."—*Gaz. Hebd.—N. Y. Med. Record.*

